# New RAD51 Inhibitors to Target Homologous Recombination in Human Cells

**DOI:** 10.3390/genes12060920

**Published:** 2021-06-16

**Authors:** Irina S. Shkundina, Alexander A. Gall, Alexej Dick, Simon Cocklin, Alexander V. Mazin

**Affiliations:** 1 Department of Biochemistry and Molecular Biology, Drexel University College of Medicine, Philadelphia, PA 19102, USA; Irina.Shkundina@jefferson.edu (I.S.S.); ad3474@drexel.edu (A.D.); sc349@drexel.edu (S.C.); 2 IDbyDNA, Bothell, WA 89011, USA; aagall@outlook.com; 3 Department of Biochemistry and Structural Biology, University of Texas Health Science Center, San Antonio, TX 78229, USA

**Keywords:** small-molecule inhibitors, DNA repair, homologous recombination, triple-negative breast cancer

## Abstract

Targeting DNA repair proteins with small-molecule inhibitors became a proven anti-cancer strategy. Previously, we identified an inhibitor of a major protein of homologous recombination (HR) RAD51, named B02. B02 inhibited HR in human cells and sensitized them to chemotherapeutic drugs *in vitro* and *in vivo*. Here, using a medicinal chemistry approach, we aimed to improve the potency of B02. We identified the B02 analog, B02-isomer, which inhibits HR in human cells with significantly higher efficiency. We also show that B02-iso sensitizes triple-negative breast cancer MDA-MB-231 cells to the PARP inhibitor (PARPi) olaparib.

## 1. Introduction

Genomic stability depends on the timely repair of DNA damage caused by various exogenous or endogenous factors, such as chemical agents, UV- and ionizing radiation, or reactive products of metabolism [1,2]. Homologous recombination (HR) is an essential and evolutionarily conserved DNA repair pathway [3,4]. HR is especially important for the repair of DNA double-strand breaks (DSB) and inter-strand crosslinks (ICL), the most harmful types of DNA lesions [5].

RAD51 recombinase is the major HR protein in eukaryotes that promotes DNA strand exchange, the critical step of HR [6,7]. RAD51 forms nucleoprotein filaments on single-stranded DNA (ssDNA) [8,9], which is generated by the exonucleolytic recession of DSB ends or collapsed replication forks [10,11]. The RAD51 filament formation is assisted by a tumor suppressor protein, BRCA2, which physically interacts with RAD51, stimulates RAD51 loading onto RPA-covered ssDNA and fosters its preferential binding to ssDNA versus dsDNA [12,13,14,15]. The nucleoprotein filament then promotes the search for homology and strand exchange between the ssDNA of the filament and the homologous strand of dsDNA [7,16]. The product of DNA strand exchange, known as the D-loop, serves as a primer and a template for DNA synthesis during DSB repair [17,18].

HR operates during and after DNA replication using homologous dsDNA in sister chromatids as a template for DSB repair [19]. Therefore, its function is critical in actively proliferating cancer cells that undergo frequent replication cycles. Consistent with that, RAD51 was shown to be overexpressed in different types of cancer, including triple-negative breast cancer [20,21,22]. Increased RAD51 levels are associated with chemotherapy and radiotherapy resistance and poor prognosis for survival [23]. RAD51 depletion leads to tumor cell sensitization to chemotherapy [24] and can slow tumor growth [25]. Moreover, RAD51 knockdown has been shown to reduce breast cancer metastasis in mouse models [26].

RAD51 is an important target for the development of novel anti-cancer therapies [27,28]. In addition to sensitizing cancer cells to traditional radiation- and chemotherapies, RAD51 inhibitors (RAD51i) may also render tumors proficient in HR, responsive to inhibitors of poly (ADP ribose) polymerase (PARPi) that generally target HR-deficient cancers, e.g., cancers carrying mutated BRCA1/2 genes [29]. Several small-molecule RAD51 inhibitors have been developed to increase cancer cell sensitivity to chemo- and radiotherapy [30]. The RAD51i of the RI series interferes with RAD51 multimerization [31,32]. The IBR series inhibitors also disrupt RAD51 multimerization as well as its interaction with BRCA2, which is important for RAD51 loading on RPA-covered ssDNA *in vivo* [33,34]. Recently, a new series of inhibitors disrupting the BRCA2–RAD51 interaction interface has been found through virtual screening with subsequent testing [35].

Previously, our laboratory identified RAD51i B02 [36,37,38]. Here, to improve the potency of B02, we performed structure–activity relationship (SAR) analysis that resulted in the development of a new series of B02 analogs, B02-isomer (B02-iso) and its derivates. We show that these new compounds are significantly more efficient than B02 in inhibiting HR in human cells. We also demonstrate that HR deficiency induced by B02-iso in triple-negative breast cancer MDA-MB-231 cells sensitizes these cells to the PARP inhibitor (PARPi) olaparib. Using surface plasmon resonance (SPR), we show that the B02-iso series compounds bind RAD51 with a higher affinity than B02. We suggest that these new compounds represent an important step towards the development of HR-targeting cancer therapies.

## 2. Materials and Methods

### 2.1. Chemicals

B02 phenyl group derivatives were purchased from Hit2Lead. Olaparib was purchased from Selleckchem (Houston, TX, USA). B02-iso, its derivatives and p-formyl-benzyl derivative of B02 were synthesized in this study. The general method of synthesis of the compounds *6 a-g* (B02-iso and derivatives) is shown in Figure S4 and described fully in the supplementary methods. Briefly, intermediates of B02-iso synthesis were obtained by a general procedure published in [39,40]. P-formyl-benzyl derivative of B02 (Figure S1, *compound 10,* Figure S3) was synthesized from the *intermediates 7,8,9* (Figure S3). *Intermediate 7* was obtained according to the procedure described in [41]. Reaction mixtures and isolated compounds were analyzed using reversed-phase HPLC on a Gemini^®^ 5 µm C18 column (10 × 2 mm, Phenomenex), and elution was performed with a linear gradient of acetonitrile in 0.1% formic acid (for [M + H]^+^ signals) and in 0.1 M ammonium formate (for [M − H]^+^ signals). LC-MS data were obtained by electrospray ionization (ESI) on Agilent 1200 series (LC/MSD Trap XCT Plus). Proton NMR spectra were obtained on a Bruker Biospin 400 instrument. NMR samples were prepared in DMSO-d6, and residual protonated solvent was used as an internal chemical shift standard. All compounds were dissolved in DMSO and stored at −80 °C.

### 2.2. Cell Culture

MDA-MB-231, U-2 OS DR-GFP, and U-2 OS IndDR-GFP cells were cultured in complete DMEM media (Sigma-Aldrich, St. Louis, MO, USA) containing 10% fetal bovine serum (Gibco, Waltham, CA, USA), 100 units/mL penicillin, and 100 µg/mL streptomycin. For the culturing of U-2 OS IndDR-GFP cells, 80 µg/mL G418 was additionally added into the media. MCF 10A cells were grown in DMEM/F-12 media (Gibco, Waltham, CA, USA) containing 5% horse serum (Invitrogen, Waltham, CA, USA), 20 ng/mL epidermal growth factor (EGF, Sigma, St. Louis, MO, USA), 10 µg/mL insulin (Invitrogen, Waltham, CA, USA), 0.5 µg/mL hydrocortisone (Sigma, St. Louis, MO, USA), 100 ng/mL cholera toxin (Sigma, St. Louis, MO, USA), 100 units/mL penicillin (Sigma, St. Louis, MO, USA) and 100 µg/mL streptomycin (Sigma, St. Louis, MO, USA). For transfections, Opti-MEM (Gibco, Waltham, CA, USA) and antibiotic-free DMEM were used. All cell lines were cultured in a humidified atmosphere containing 5% CO_2_ at 37 °C.

### 2.3. Surface Plasmon Resonance (SPR) Assay

The binding of RAD51i to RAD51 was examined using a ProteOn XPR36 SPR Protein Interaction Array System (Bio-Rad, Hercules, CA, USA) at 25 °C. ProteOn GLH sensor chips were preconditioned with two 10 s pulses of 50 mM NaOH, 100 mM HCl, and 0.5% SDS followed by the system equilibration with the running buffer (20 mM sodium phosphate, 150 mM NaCl, and 0.005% Tween 20, pH 7.4). The surface of a GLH sensorchip was subsequently activated with a 1:100 dilution of a 1:1 mixture of 0.2 M EDC and 0.05 M Sulfo-NHS. Purified HsRAD51 was diluted to 500 µg/mL in 10 mM sodium acetate, pH 5.5, and injected immediately after chip activation across the ligand flow channels at 30 µL/min for 5 min. Unreacted protein was washed out, and the excess of unreacted ester groups on the sensor surface was capped by an injection of 1 M ethanolamine-HCl, pH 8.0, at 5 µL/min for 5 min. A reference surface to correct for nonspecific binding was similarly created by immobilizing an IgG b12 anti-HIV-1 gp120 antibody (IgG b12 anti-HIV-1 gp120; was obtained through the NIH AIDS Reagent Program, Division of AIDS, NIAID, NIH: Anti-HIV-1 gp120 Monoclonal (IgG1 b12) from Drs. Dennis Burton and Carlos Barbas). Serial dilutions of the compounds were prepared in the running buffer supplemented with 8% DMSO and injected at a flow rate of 100 μL/min for a 1 min association phase, followed by a 5 min dissociation phase using the “one-shot kinetics” functionality of the ProteOn instrument. Data were analyzed with ProteOn Manager Software version 3.0 (Bio-Rad; Hercules, CA, USA). The responses from the reference flow cell were subtracted to account for the non-specific binding and injection artifacts. Experiments were repeated at least three times. The equilibrium dissociation constants (K_D_) for the interactions were calculated in ProteOn Manager Version 3.1.0.6 (Bio-Rad; Hercules, CA, USA) using the equilibrium analysis function.

### 2.4. Docking Simulations

The PDB files for B02, B02-iso, m-Br-B02-iso, and p-I-B02-iso were prepared and then energy-minimized using Flare version 4 (Cresset^®^, Litlington, Cambridgeshire, UK,) with a root mean squared (RMS) gradient cutoff of 0.2 kcal/mol/A and 10,000 iterations. The crystal structure of human Rad51-ATP filament (PDB code: 5NWL) was prepared using Flare, version 4 (Cresset^®^, Litlington, Cambridgeshire, UK) to allow for protonation at pH 7.0 and the removal of residue gaps. A dimeric unit of the pre-prepared human Rad51-ATP filament structure was further prepared using Autodock tools [42], where essential hydrogen atoms, Kollman united atom type charges, and solvation parameters were added. The grid box for the docking search was centered around the entire hRad51 dimer with a spacing grid of 0.375 Å using the Autogrid program [42]. Docking calculations were performed using AutoDock via DockingServer [43]. B02, B02-Isomer, m-Br-B02-Isomer, and p-I-B02-Isomer were further energy minimized using the MMFF94 Force Field method and Gasteiger partial charges added. Non-polar hydrogen atoms were merged, and rotatable bonds were defined. AutoDock parameter set- and distance-dependent dielectric functions were used in the calculation of the van der Waals and the electrostatic terms, respectively. Docking simulations were performed using the Lamarckian genetic algorithm (LGA) and the Solis and Wets local search method [44]. Initial position, orientation, and torsions of the ligand molecules were set randomly. Rotatable torsion angles were released during docking. Each docking experiment was derived from 100 different runs that were set to terminate after a maximum of 2,500,000 energy evaluations. The population size was set to 150. During the search, a translational step of 0.2 Å and quaternion and torsion steps of 5 were applied.

### 2.5. Construction of U-2 OS IndDR-GFP Cell Line

Exponentially growing U-2 OS DR-GFP cells were seeded at 2 × 10^5^/well into 6-well plates containing DMEM supplemented with 100 units/mL penicillin and 100 µg/mL streptomycin. The next day, the media were removed, cells were incubated in antibiotic-free DMEM for 2 h at 37 °C. Transfection mixtures were prepared by adding 250 µL of Opti-MEM medium (Gibco, Waltham, CA, USA) combined with 3.6 µL of Lipofectamine 2000 (Invitrogen, Waltham, CA, USA) to 250 µL of Opti-MEM medium containing 1 µg DNA of ddSceGR plasmid, expressing chimeric inducible I*-Sce*I endonuclease [45]. After 20 min of incubation at room temperature, 500 µL of transfection mixtures were added to 1 mL of antibiotic-free DMEM freshly added into each well and incubated for 3 h at 37 °C. Then, transfection media were removed, cells were washed with PBS buffer (137 mM NaCl, 2.7 mM KCl, 8 mM Na_2_HPO_4_, and 2 mM KH_2_PO_4_), DMEM containing 100 units/mL penicillin and 100 µg/mL streptomycin was added, and incubation continued. The next day, G418 antibiotic (Sigma, St. Louis, MO, USA) at 0.8 mg/mL was applied for 4 days to select for the cells that integrated the ddSceGR plasmid into the genome. After completion of selection, cells were trypsinized, and 2.5 × 10^3^ cells were seeded into a 150 mm cell culture dish with DMEM containing 0.2 mg/mL G418, 100 units/mL penicillin, and 100 µg/mL streptomycin and grown for 2 weeks. Individual colonies were observed under a microscope, and their position was marked with a marking pen on the bottom of the dish. The growth medium was removed, and the dish was washed twice with PBS buffer. After PBS was removed, autoclaved Corning Cloning Cylinders (Thermo Fisher Scientific, Waltham, MA, USA) were set over individual colonies using sterile vacuum grease. A total of 0.1 mL of 0.25% Trypsin was added into each cloning cylinder, and the plate was incubated for 5 min at 37 °C. Then, 0.25% Trypsin was neutralized with an equal volume of media inside of each cloning cylinder. After trypsinization, cells from each cylinder were seeded into individual wells of 12-well plates. Individual clones were further expanded in 100 mm cell culture dishes in the presence of 80 µg/mL G418. Every clone was tested for formation of the GFP-positive cells indicative of the HR activity using flow cytometry either after induction of chimeric I*-Sce*I in the presence of Shield1 (Clontech Laboratories; Mountain View, CA, USA) and triamcinolone acetonide (Sigma-Aldrich; St. Louis, MO, USA) or without induction (see DR-GFP assay section below). The clone that gave the most significant difference in the formation of GFP-positive cells between I*-Sce*I induced and uninduced states was named U-2 OS IndDR-GFP and used for all inhibitor screening.

### 2.6. Inducible I-SceI Endonuclease DR-GFP Assay (IndDR-GFP)

Exponentially growing U-2 OS IndDR-GFP cells were seeded at a density of 2 × 10^5^ cells/well into 6-well plates with DMEM media containing 80 µg/mL G418 and indicated concentrations of RAD51i(s). For I*-Sce*I induced samples, the media were supplemented with 0.5 µM Shield1 (Clontech, Mountain View, CA, USA) with 100 nM triamcinolone acetonide (Sigma, St. Louis, MO, USA). After 24 h of incubation, the media were removed, cells were washed with the DMEM, and the incubation continued for 48 h in DMEM containing 100 units/mL penicillin, 100 µg/mL streptomycin, and 80 µg/mL G418. Then, cells were trypsinized, trypsin solution was neutralized with an equal volume of media, and 10% formaldehyde solution in PBS was added for fixation to reach a final concentration of formaldehyde of 3.3%. The yield of GFP-positive cells indicative of HR activity was measured by flow cytometry with the Guava easyCyte system, and the data were analyzed with the InCyte program (Millipore; Burlington, VT, USA).

### 2.7. Cell Viability Assay

For all cell viability experiments, except experiments involving MMS treatment, cells were seeded into the media containing indicated concentrations of tested compounds or corresponding amounts of DMSO in controls. The concentration of DMSO in media was 0.1%. MDA-MB-231 cells were seeded into 24-well plates at 3.5 × 10^4^ cells/well for cells for a 4-day treatment or at 5000 cells/well for a 10-day treatment. MCF 10A cells were seeded into 24-well plates at 1.5 × 10^4^ cells/well for a 4-day treatment or at 500 cells/well for a 10-day treatment. In the case of 10-day treatments, the media with compounds were renewed twice on the 4th and the 7th days of treatments. After completion of the treatments, cells were harvested, and their viability was determined by flow cytometry with Guava ViaCount reagent (Luminex; Austin, TX USA). Data were analyzed using the Guava ViaCount software (Millipore; Burlington, VT, USA). The IC_50_ values were calculated using the GraphPad Prism 5 software (San Diego, CA, USA).

For experiments in which MDA-MB-231 cells were treated with MMS, cells were seeded in 24-well plates at 3.5 × 10^4^ cells/well. The next day, cells were treated with 100 µM MMS (Sigma, St. Louis, MO, USA) for 1 h in the presence of increasing concentrations of olaparib and 2 µM B02-iso where indicated. Then, cells were washed twice with PBS, which was followed by incubation with olaparib at indicated concentrations and 2 µM B02-iso where indicated for 4 days. After completion of the treatments, cells were harvested, and their viability was determined by flow cytometry using Guava ViaCount reagent (Luminex; Austin, TX, USA). Data were analyzed using the Guava ViaCount software (Millipore; Burlington, VT, USA).

### 2.8. RAD51 Foci Formation

U-2 OS IndDR-GFP cells were seeded into 8-well polystyrene vessel culture slides at 5 × 10^4^ cells/well. The next day, cells were pretreated with B02-iso or 0.1% DMSO (for control cells) for 1 h, followed by the treatment with 5 µM cisplatin for 4 h or left untreated (for control cells). After 4 h of incubation, cells were placed on ice, washed with PBS buffer (twice, 5 min each), pretreated with 0.5% Triton X-100 for 5 min, washed with PBS (twice, 5 min each). Cells were removed from ice and fixed with 4% methanol-free formaldehyde (Pierce; Waltham, CA, USA) in PBS for 10 min. Cells were washed with PBS (twice, 5 min each), placed on ice, and permeabilized with 1% Triton X-100 in PBS for 10 min. Cells were removed from ice, washed with PBS (once, 5 min), blocked with a blocking buffer (5% BSA (Bovine Serum Albumin) in PBS) for 30 min, and incubated with rabbit polyclonal RAD51 antibody (1:1000, GeneTex, GTX100469) and mouse monoclonal anti-phospho-histone H2A.X (Ser 139) antibody (1:20,000, clone JBW301, Millipore 05-636) in blocking buffer, containing 0.3% Triton X-100 overnight at 4 °C. Next, cells were washed with PBS (twice, 5 min each), incubated at room temperature in the dark with the secondary Alexa Fluor 488 conjugated anti-rabbit antibody (1:5000, Invitrogen, A-11034) and Alexa Fluor 568 conjugated anti-mouse antibody (1:1000, Invitrogen, A-21124) for 1 h. Cells were washed with PBS (3 times, 5 min each); afterwards, the polystyrene vessel was removed from the slide, and cells were covered with Prolong Gold Antifade Reagent with DAPI (Invitrogen; Waltham, CA, USA) and mounted with a 24 × 50 mm coverslip. Images were acquired with Evos FL Auto Imaging System (Life Technologies; Carlsbad, NM, USA). For foci quantification, 50 cells were chosen for each treatment condition at random, and foci number was counted using ImageJ software. Cells bearing more than 10 RAD51 or 10 γ-H2A.X foci per nucleus were considered foci positive. The percent of foci-positive cells was averaged from 3 biological replicates.

### 2.9. Preparation of Nuclear and Whole-Cell Extracts

For the preparation of the nuclear extracts, exponentially growing U-2 OS IndDR-GFP cells were seeded at 1 × 10^6^ cells per dish in 100 mm dishes (3 for each sample) with DMEM, containing 100 units/mL penicillin, 100 µg/mL streptomycin, 80 µg/mL G418, and, when indicated, 0.5 µM Shield1 (Clontech Laboratories; Mountain View, CA, USA) with 100 nM triamcinolone acetonide (Sigma, St. Louis, MO, USA). After 24 h, the media were removed, cells were washed with DMEM, and incubation continued for 48 h in DMEM containing 100 units/mL penicillin, 100 µg/mL streptomycin, and 80 µg/mL G418. Cells were harvested and washed twice by resuspending cell pellets in cold PBS with subsequent centrifugation at 500× *g* for 5 min at 4 °C. Nuclear extracts were obtained from the cell pellets using NE-PER Nuclear and Cytoplasmic Extraction reagents (Thermo Fisher Scientific; Waltham, CA, USA).

For the preparation of the whole-cell extracts, MDA-MB-231 cells grown in DMEM were harvested, cell pellets were placed on ice, washed twice in cold PBS, followed each time with centrifugation at 500× *g* for 5 min. Cell pellet volumes were visually evaluated and resuspended in a cold lysis buffer containing 25 mM Tris-HCl, pH 8.0, 120 mM NaCl, 1 mM EDTA, 0.5% NP-40, and 1 tablet of cOmplete Protease Inhibitor Cocktail (Roche; Basel, Switzerland) per 50 mL of buffer. The lysis buffer volume was set as 10× of the volume of a cell pellet for every sample. Cell suspensions were mixed by vigorous vortexing for 5 s and incubated on ice for 30 min. During incubation, cells were vigorously vortexed for 5 s every 10 min. Then, cell extracts were spun down at 16,000× *g* for 15 min at 4 °C, and supernatants were transferred to fresh Eppendorf tubes.

### 2.10. Western Blotting

Protein concentrations in the nuclear and whole-cell extracts were determined by Bradford assay (Bio-Rad), and the equivalent protein amounts were subjected to electrophoresis in 12% SDS-Polyacrylamide gels (29:1) in Tris-Glycine-SDS buffer. After gel-electrophoresis, proteins were transferred to PVDF membranes (Amersham, Piscataway, NJ, USA) using Mini Trans-Blot Cell (BioRad, Hercules, CA, USA) at 80V for 40 min. PVDF membranes were washed in TBS and blocked with a blocking buffer (5% nonfat dry milk in TBS-T) for 1 h. Anti-RAD51 rabbit polyclonal antibody (1:1000, GeneTex, GTX100469), anti-FKBP12 monoclonal mouse antibody (1:100, BD Biosciences; Franklin lakes, NJ, USA, 610808) for the detection of ddSceGR, and anti-nucleolin mouse monoclonal antibody (1:1000, Santa Cruz Biotechnology; Dallas, Texas, sc-17826) were applied overnight in a blocking buffer at 4 °C. Membranes were washed twice in TBS-T (50 mM Tris-HCl, pH, 7.6, 150 mM NaCl, 0.1% Tween 20) and probed with peroxidase-conjugated AffiniPure goat anti-rabbit or anti-mouse IgG (1:10,000, Jackson ImmunoResearch; West Grove, PA, USA, 111-035-003 and 115-035-003, respectively) for 1 h. Signal was detected with SuperSignal West Pico Plus chemiluminescent substrate (Thermo Scientific; Waltham, CA, USA) with a G:B0X6 imaging system (Syngene; Bangalore, India). Band intensities were quantified with the GeneTools software (Syngene; Bangalore, India).

### 2.11. EdU Cell Proliferation Assay

Exponentially growing MDA-MB-231 and MCF 10A cells were seeded into 6-well plates at a density of 1.5 × 10^5^ cells/well in the presence of indicated concentrations of B02-iso or 0.1% DMSO (for control cells). After 24 h of incubation, cells were labeled with 10 µM Edu (Invitrogen; Waltham, CA, USA) for 1 h or left unlabeled in the control. Cells were harvested and centrifuged at 500× *g*, 4 °C for 5 min, and cell pellets were washed with PBS supplemented with 1% BSA, spun down, and fixed with 4% methanol-free formaldehyde (Pierce) in PBS for 15 min. Cells were washed with PBS containing 1% BSA, centrifuged 500× *g* for 5 min. Cell pellets were resuspended in PBS containing 0.1% Triton X-100 and incubated for 15 min for permeabilization. Cells were spun down, resuspended in freshly prepared Edu click labeling solution (1 mM CuSO_4_, 1.25 µM Alexa Fluor 488 Azide (Invitrogen; Waltham, CA, USA), 50 mM ascorbic acid in PBS) and incubated for 30 min in the dark. Cells were washed twice with PBS containing 0.1% Triton X-100, resuspended in the same buffer, and analyzed for Edu incorporation by flow cytometry using the Guava easyCyte system (Millipore; Burlington, VT, USA) with the InCyte software (Millipore; Burlington, VT, USA).

### 2.12. Cell Cycle Analysis

Exponentially growing MDA-MB-231 and MCF 10A cells were seeded into 6-well plates at a density of 1.5 × 10^5^ cells/well in the presence of indicated concentrations of B02-iso or 0.01% DMSO (for control cells). After 24 h of incubation, cells were harvested, placed on ice, and washed once with cold PBS. Cells were counted with an automated cell counter (Bio-Rad; Hercules, CA, USA), and 1 × 10^5^ cells from each sample were taken for further analysis. Cells were resuspended in PBS, and then cold, absolute ethanol was added dropwise to a cell suspension to reach a final concentration of 70%, followed by incubation on ice for 1 h. Cells were washed in PBS, resuspended in staining buffer (50 µg/mL propidium iodide, 100 µg/mL RNase A, 0.1% Triton X-100 in PBS) and incubated for 15 min in the dark at 37 °C. Cells were analyzed for DNA content by flow cytometry using the Guava easyCyte system (Millipore; Burlington, VT, USA) with the InCyte software (Millipore; Burlington, VT, USA).

### 2.13. Statistical Analysis

The GraphPad Prism 5 software was used for statistical analysis. A two-tailed t-test was applied to determine the *p*-value of the difference in the yield of GFP-positive cells in the induced and uninduced cultures of U-2 OS IndDR-GFP cells. The IC_50_ values for viability assays were determined with a variable slope nonlinear regression algorithm. To determine *p*-values for generated RAD51 and γ-H2A.X foci, one-way ANOVA with post-hoc Tukey test was used. For the comparison of 2 cell lines in Edu cell proliferation, two-way ANOVA with Bonferroni posttests was applied. One-way ANOVA with Dunett post-hoc test was used for cell cycle analysis.

## 3. Results

### 3.1. Development of an Inducible I-SceI DR-GFP Assay

**Figure 1:**
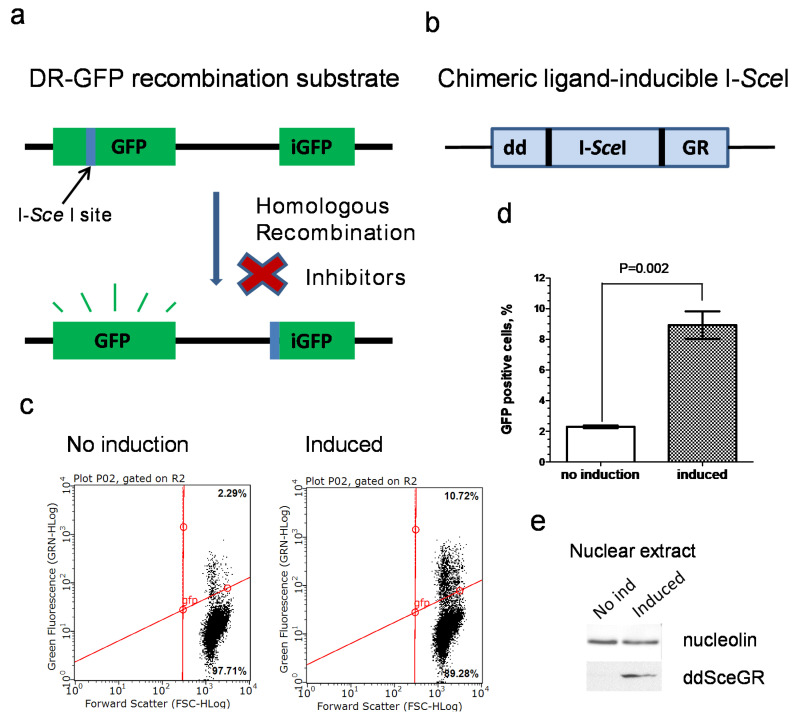
Development of an IndDR-GFP system. The schemes (**a**) of the DR-GFP reporter operation and (**b**) of the chromosomally integrated ligand-inducible I-*Sce*I. (**c**) Representative flow cytometry plots for GFP fluorescence in the DSB-induced and uninduced U-2 OS IndDR-GFP cells (clone #3). (**d**) Histogram depicting the GFP+ fractions in DSB-induced and uninduced U-2 OS IndDR-GFP cells (clone #3). (**e**) The expression level of ddSceGR in the nucleus before and after induction in U-2 OS IndDR-GFP cells (clone #3). GFP—green fluorescent protein; iGFP—internal fragment of GFP; I-*Sce*I—rare cutting endonuclease from S. cerevisiae; dd—destabilizing domain of FKBP12; GR—the ligand binding domain of the rat glucocorticoid receptor.

To examine the effect of new B02 analogs on HR in human cells, we utilized the DR-GFP system. The canonical DR-GFP system is commonly used to measure the frequency of DSB-induced HR in human (mammalian) cells [46]. In this system, a functional copy of the GFP gene is reconstituted by DSB-induced HR from two inactive GFP copies integrated into the chromosome (Figure 1a). However, the induction of the recombination event requires transfection of the cell with a plasmid encoding I*-Sce*I endonuclease that generates a DSB in one of the inactive GFP copies. To examine multiple compounds, it was important to eliminate the time- and labor-consuming step of transfection. Therefore, we decided to modify the DR-GFP system by integrating the coding sequence of ligand-inducible I*-Sce*I endonuclease [45] into the chromosome of U-2 OS cells bearing the DR-GFP construct.

We used a DNA construct encoding chimeric ligand-inducible I*-Sce*I endonuclease consisting of I*-Sce*I sequence [45] fused with the sequence of FKBP12 destabilizing domain (dd) on the N-terminus and with the sequence of rat glucocorticoid receptor (GR) ligand-binding domain on the C-terminus (Figure 1b). The modified fusion protein was named ddSceGR. The GR ligand-binding domain confers the translocation of ddSceGR to the nucleus upon the binding of the synthetic ligand triamcinolone acetonide. The destabilizing domain of FKBP12 helps to maintain the expression level of ddSceGR low in the absence of the ligand Shield1 that blocks the effect of the FKBP12 destabilizing domain, thus increasing the expression level of ddSceGR [45]. In this way, a fully functional endonuclease is produced and exported to the nucleus upon the addition of the two chemical ligands to a growth medium, which replaces the tedious procedure of cell transfection.

We chose to integrate a ddSceGR-expressing construct into the U-2 OS DR-GFP cell line. U-2 OS DR-GFP cells were transfected with the ddSceGR encoding plasmid [45]. Transfection was followed by antibiotic selection with G418 and subsequent isolation of G418-positive clones. In total, 16 G418-positive clones were isolated and analyzed for their HR repair activity in the absence and in the presence of Shield1 and triamcinolone acetonide. In total, 2 of the 16 G418-positive clones (#3 and #11) were found to significantly increase the level of HR in the presence of the ligands. The induction of ddSceGR led to a four-fold increase in HR in clone #3 cells measured by flow cytometry (Figure 1c,d). Figure 1e shows the level of ddSceGR in the nucleus of clone #3 after 24 h of incubation with triamcinolone acetonide and Shield1. Clone #3 was named the U-2 OS Inducible DR-GFP (IndDR-GFP) cell line and was used further for compound screening in subsequent experiments. To validate the accuracy of a new IndDR-GFP system, B02 compound was tested several times in both U-2 OS DR-GFP and U-2 OS IndDR-GFP assays; the IC_50_ values of HR inhibition were 17.9 ± 3.5 µM and 17.7 ± 3.9 µM in these two assays, respectively.

### 3.2. Testing Activity of New B02 Derivatives

**Figure 2:**
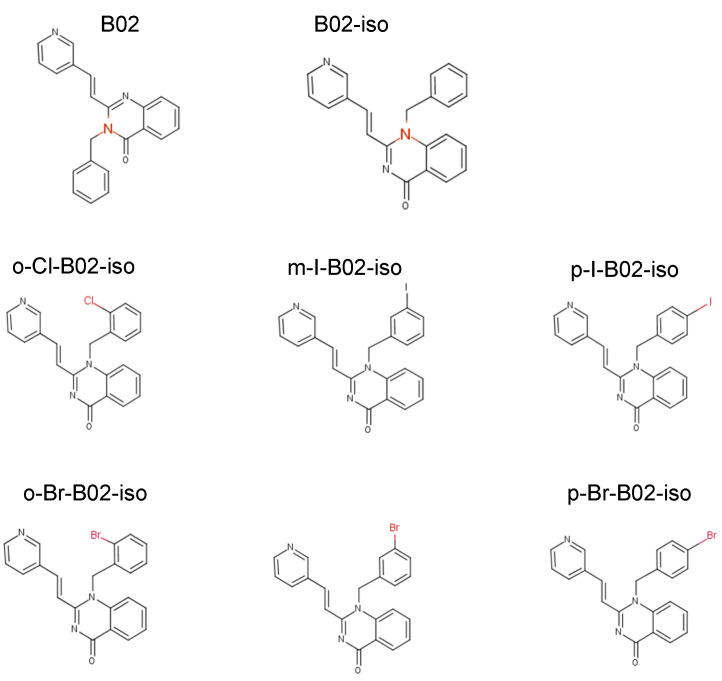
Chemical structures of B02, B02-iso and halogen-containing derivatives of B02-iso.

To improve the potency of the B02-series [36], we first examined several commercially available B02 analogs for their ability to inhibit HR in human U-2 OS cells using the IndDR-GFP assay. B02 contains three chemical groups: quinazoline, pyridyl, and a benzyl moiety (Figure 2). We [36], among others [47], previously showed that modifications of pyridyl and quinazoline groups dramatically reduce the B02 anti-HR activity. Here, we tested the effect of the replacement of the benzyl group with the phenyl group derivatives on the potency of inhibition of HR in human cells. Four subgroups of B02 analogs were tested (Figure S1): phenyl halogen derivatives (p-bromophenyl, p-chlorophenyl, o-dichlorophenyl), methoxyphenyl derivatives (o-methoxyphenyl, m-methoxyphenyl, p-methoxyphenyl), hydroxyphenyl derivatives (p-hydroxyphenyl, p-hydroxyethyl-phenyl), and methylphenyl derivatives (o-methylphenyl, o-methylphenyl(I)). We also tested the effect of a substitution of the benzyl group of B02 by the p-formyl-benzyl group yielding in p-formyl benzyl derivative (Figure S1, the synthesis scheme is shown on Figure S3). We found that all tested compounds with a modified phenyl/benzyl group displayed a lower anti-HR activity compared to the parental B02 (Figure S2).

**Figure 3:**
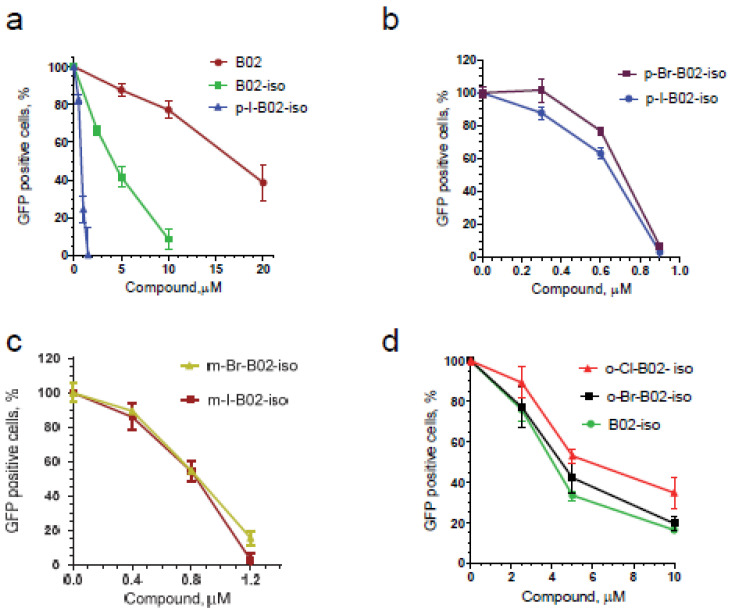
B02-iso and its halogen derivatives efficiently inhibit HR in human U-2 OS cells. The efficiency of HR was measured using IndDR-GFP assay. The effect of the following compounds was examined: (**a**) B02, B02-isomer and p-I-B02-iso; (**b**) p-Br-B02-iso and p-I-B02-iso; (**c**) m-Br-B02-iso and m-I-B02-iso; (**d**) o-Cl-B02-iso, o-Br-B02-iso and B02-iso. The experiments were repeated at least three times. The error bars represent the standard error of the mean (SEM).

Next, we studied the effect of the position of the benzyl group on the quinazoline ring. While in B02 the benzyl group is attached to the N3 nitrogen of the quinazoline ring, we synthesized B02 isomer (B02-iso), in which the benzyl group was attached to the N1 nitrogen (Figure 2; the synthesis scheme is shown in Figure S4). Using the IndDR-GFP assay, we found that the B02-iso inhibited HR in U-2 OS cells considerably stronger than B02 (Figure 3a). The IC_50_ of B02-iso was 4.3 µM, whereas the IC_50_ of B02 was 17.7 µM (Table 1). Thus, changing the position of the benzyl group in the quinazolinone moiety improved the potency of the compound approximately four-fold.

We next investigated if modifications at the ortho, meta or para position of this benzyl moiety would impact the compound potency. We, therefore, synthesized six halogen derivatives of B02-iso, with halogen atoms attached to the ortho, meta, or para positions of the benzyl ring (Figure 2) and tested their activity using the IndDR-GFP assay. The most active compound among those halogen derivatives of B02-iso was the para-I-B02-iso, with an IC_50_ of HR inhibition of 0.72 µM (Figure 3a,b, Table 1). Para-Br-B02-isomer was almost as active as para-I-B02-isomer with the IC_50_ of 0.80 µM (Figure 3b, Table 1). However, para-Br-B02-isomer had low solubility; even in a 100% DMSO, its maximal soluble concentration was 0.5 mM. Meta-I-B02-iso and meta-Br-B02-iso were slightly less potent HR inhibitors with IC_50_s of 0.86 and 0.90 µM, respectively (Figure 3c, Table 1). The least potent halogen derivatives were ortho-Br-B02-iso and ortho-Cl-B02-iso with IC_50_ values of 4.60 and 5.56 µM, respectively (Figure 3d, Table 1), both higher than the IC_50_ of B02-iso. Overall, B02-iso derivatives with halogen substituents in para-position of the benzyl ring were the most active HR inhibitors among all tested B02-iso series compounds.

### 3.3. B02-iso Analogs Bind Directly to RAD51 In Vitro

Previously, employing surface plasmon resonance (SPR), we have shown that B02 binds to RAD51 directly *in vitro* [37]. Here, we use SPR to examine the binding of B02-iso and its analogs to RAD51. RAD51 was immobilized on a chip, and serial dilutions of the compounds were flowed over the chip to test for their abilities to bind the protein. We found that B02-iso and its halogen derivates bind directly to RAD51 (Table 1). Moreover, the most active inhibitor of HR, para-I-B02-iso, showed the highest binding affinity to RAD51 (the equilibrium dissociation constant, K_D_ = 1.4 µM). However, a correlation between binding affinities of B02-iso analogs for RAD51 with their anti-HR activities in U-2 OS IndDR-GFP cells was not absolute. Thus, the K_D_(s) were identical for B02 and B02-iso (14.6 µM) (Table 1), while B02-iso showed significantly stronger anti-HR activity than B02 in human cells. These data may indicate that specific conformations of inhibitors in the complex with RAD51 may affect the inhibitor’s activity. Moreover, unequal anti-HR activities of the compounds with identical K_D_(s) may be caused by different cell permeabilities of the compounds.

**Figure 4:**
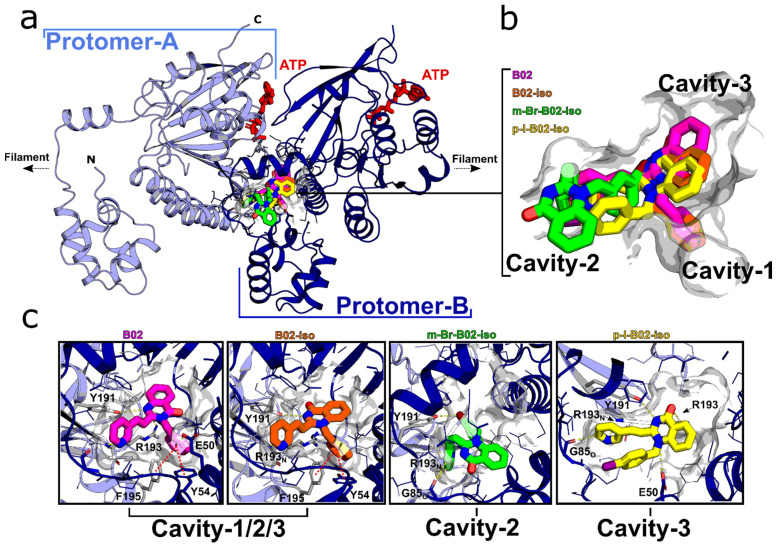
Docking of B02, B02-iso and B02-iso analogs to human RAD51 protein. (**a**) Dimeric unit of the RAD51-ATP filament (PDB code: 5NWL) and docked poses of B02 (pink), B02-iso (orange), m-Br-B02-iso (green) and p-I-B02-iso (yellow) within the protomer–protomer interface; ATP is shown in red. (**b**) Close-up view of the binding site with three distinct cavities, cavity-1, -2 and -3. (**c**) Molecular details of compound stabilization within the three cavities (shown in light gray surface); polar contacts are highlighted with yellow dashed lines, while putative π-π interactions (B02, B02-iso) are indicated with red dashed lines; an O- or N- in the index of a residue highlights the contribution of a mainchain carbonyl or nitrogen atom for the interaction, respectively; residues contributing polar contacts are highlighted with an increased stick radius in light blue/gray (Protomer A) or dark blue (Protomer B).

To date, no structure of RAD51 in complex with B02 is available. However, several docking studies suggest that B02 binds within the dimerization interface of a RAD51 filament or within the Walker A motif of the ATP-binding domain [47,48]. To better understand the possible structural implications of the B02-iso derivatives binding to RAD51 and the observed potency variations, we docked B02, B02-iso and its derivatives to the human RAD51 filament (pdb code: 5NWL) using a Flare/Autodock workflow (Cresset^®^, Litlington, Cambridgeshire, UK). Docking calculations were performed with the ATP molecule in the crystal structure of RAD51. The preferred B02 binding site was identified within the RAD51 dimerization interface (Figure 4a–c). B02 and the B02-iso bind within this pocket and occupy with their benzyl, pyridyl, and quinazoline moiety cavity-1, -2, and -3, respectively (Figure 4b,c). Notably, the quinazoline moiety is flipped by 180° in the B02-iso structure. Both structures display favorable π-π stacking of the benzyl moiety and Tyr54 and Phe195 and additional polar contacts with Tyr191 and Arg193 (Figure 4c). The m-Br-B02-iso occupies only cavity-2 and the p-I-B02-iso only cavity-3 in our docking poses, and the π-π-interactions are not present in both halogenated isomers. However, additional polar contacts to the Arg193 backbone nitrogen, Gly85 backbone carbonyl, and in the case of p-I-B02-iso to Glu50 are present that could potentially account for the potency variations of the B02-iso derivatives.

### 3.4. B02-iso Inhibits RAD51 Foci Formation in Human Cells

**Figure 5:**
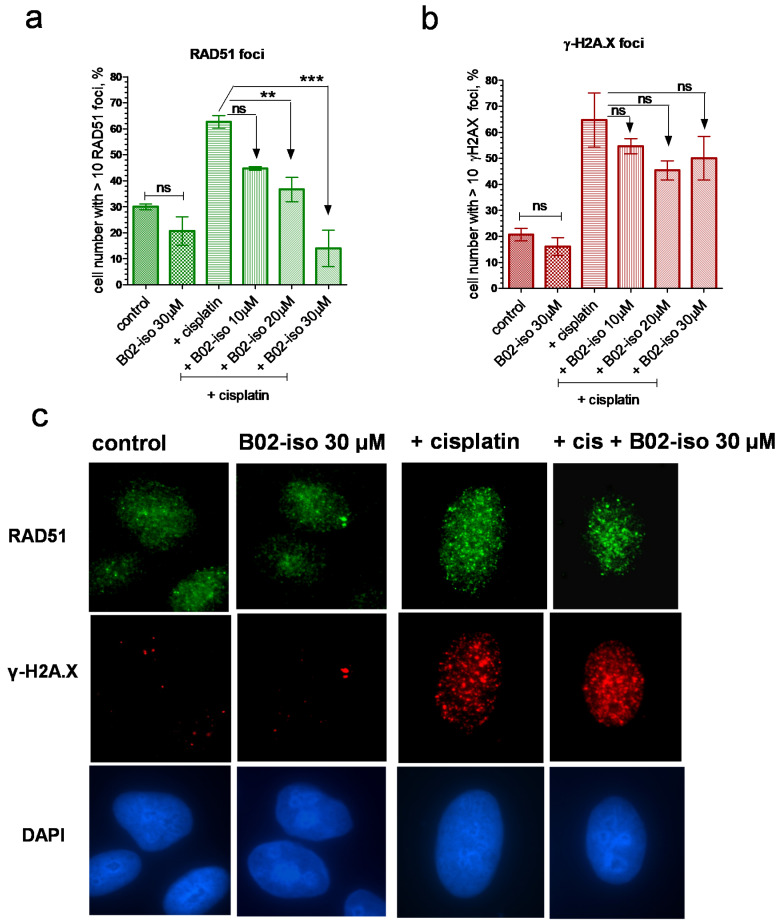
B02-iso inhibits RAD51 foci but not γ-H2A.X foci formation in U-2 OS cells in response to cisplatin treatment. (**a**) Percent of the cells bearing more than 10 RAD51 foci per nucleus in the cells either untreated (control), treated with B02-iso or with cisplatin or treated with B02-iso (in indicated concentrations) followed by treatment with cisplatin. (**b**) Percent of the cells bearing more than 10 γ-H2A.X foci per nucleus in the untreated cells (control) or treated with B02-iso or with cisplatin or with a combination of B02-iso and cisplatin as in (**a**,**c**), Typical images of the cells at indicated conditions stained with anti-RAD51 N1C2 antibody, anti-γ-H2A.X antibody and DAPI. cis—cisplatin. Error bars represent the SEM. Experiments were repeated at least 3 times. ** *p* < 0.01, *** *p* < 0.001, ns—not significant.

RAD51 forms foci in cell nuclei in response to DNA damage induced by exogenous or endogenous chemical agents or radiation [49]. RAD51 foci likely represent the sites of DNA repair in the nucleus. Disruption of RAD51 foci by inhibitors indicates their ability to interact with RAD51 in the cell and inhibit its DNA repair function. Previously, we have shown that B02 disrupts RAD51 foci formation induced by cisplatin [38]. Here, we examined whether B02-iso shares this ability. First, we found that the treatment of U-2 OS IndDR-GFP cells with B02-iso (30 µM) alone for 1 h did not significantly affect RAD51 foci formation (Figure 5a,c). Next, we found that B02-iso reduced RAD51 foci formation induced by cisplatin in a concentration-dependent manner; at 30 µM, it almost completely eliminated RAD51 foci formation. Notably, treatment with B02-iso at 4 µM for 24 h did not change RAD51 expression level (Figure S5). A relatively high B02-iso concentration required for the inhibition of foci formation compared with the inhibition of HR is likely due to a considerably shorter treatment time, 1 h vs. 24 h in the IndDR-GFP assay. Importantly, B02-iso at indicated concentrations did not reduce the level of γ-H2A.X foci (Figure 5b,c), indicating that the compound does not impair the initial steps of DSB repair.

### 3.5. B02-iso Analogs Show Preferential Antiproliferative Effect in Cancer Cells

**Figure 6:**
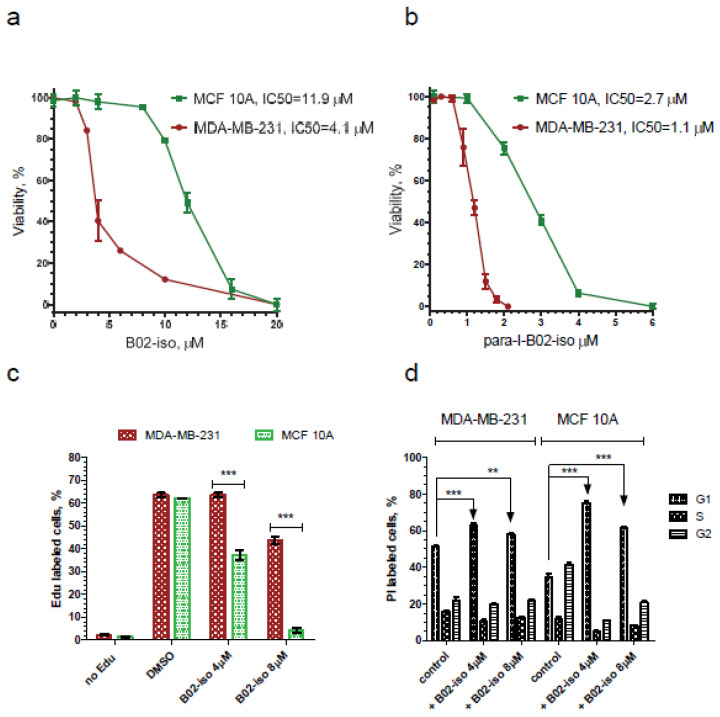
MDA-MB-231 cancer cells show greater sensitivity to B02-iso than breast epithelial MCF 10A cells. Effect of B02-iso (**a**) and para-I-B02-iso (**b**) on the viability of MDA-MB231 breast cancer cells and MCF 10A breast epithelial cells. (**c**) B02-iso halts proliferation in MCF 10A cells but not in MDA-MB-231 cells. (**d**) B02-iso causes prominent G1 cell cycle arrest in MCF 10A cells. Experiments were repeated at least 3 times, and representative graphs are shown. Error bars indicate the SEM, ** *p* <0.01, *** *p* < 0.001.

Since RAD51 is often overexpressed in cancer cells and that this overexpression correlates with poor patient survival [20,22,50], we suggested that B02-iso analogs may have a more substantial antiproliferative effect on cancer cells than on normal cells. We tested the effect of B02-iso and its para-I-B02-iso analog on the growth of triple-negative breast cancer cells, MDA-MB-231, and normal immortalized breast epithelial cells, MCF 10A. We found that B02-iso and para-I-B02-iso decreased the viability of MDA-MB-231 with an IC_50_ of 4.1 and 1.1 µM, respectively, over the course of a 4-day treatment (Figure 6a,b). MCF 10A cells showed approximately 3 times lower sensitivity to both B02-iso and para-I-B02-iso, with an IC_50_ of 11.9 and 2.7 µM, respectively (Figure 6a,b).

We suggested that the increased viability of MCF 10A cells in the presence of B02-iso analogs may be caused by the activation of a cell cycle checkpoint, which prevents them from initiating DNA replication. To test this hypothesis, we measured the fraction of DNA-replicating cells using the Edu cell proliferation assay. In untreated controls, approximately one-half of MDA-MB-231 and MCF 10A cells were undergoing DNA replication (Figure 6c). At 4 µM B02-iso, this fraction was dropped approximately two-fold in MCF 10A cells, whereas it remained unchanged in MDA-MB-231 cells. At 8 µM B02-iso, the fraction of DNA-replicating cells was abolished almost entirely in MCF 10A cells and was still present, though reduced by approximately 20%, in MDA-MB-231 cells. We conclude that breast cancer cells are less capable of cell cycle arrest than breast epithelial cells during B02-iso treatment. Cell cycle analysis confirmed prominent G1 arrest for MCF 10A cells in the presence of B02-iso (Figure 6d).

### 3.6. B02-iso Potentiates the Killing of Triple-Negative Breast Cancer Cells with Olaparib

**Figure 7:**
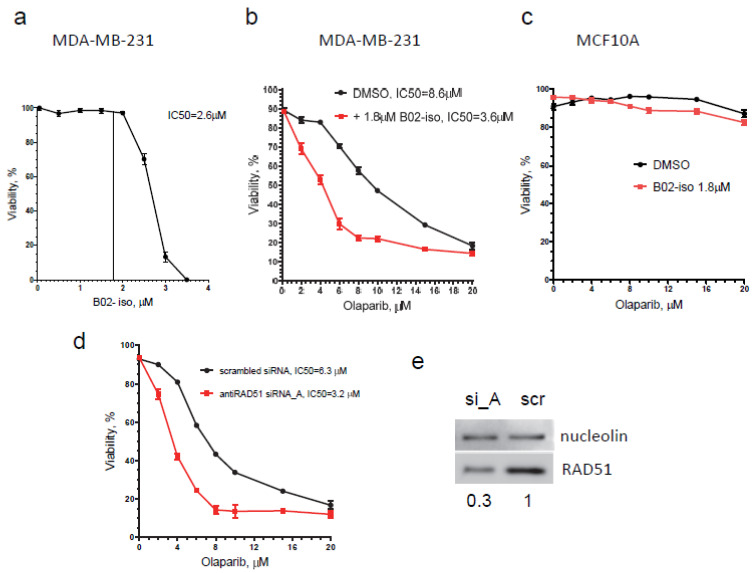

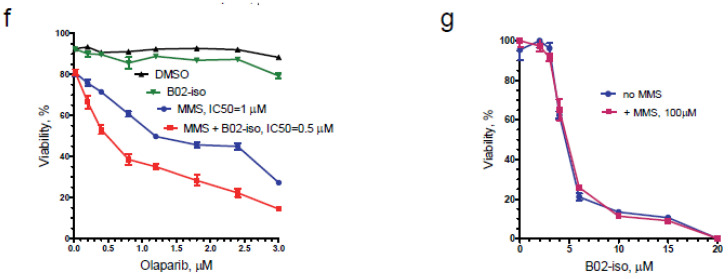
B02-iso potentiates the effect of olaparib on MDA-MB-231 cells. (**a**) Viability of MDA-MB-231 cells treated with B02-iso for 10 days; (**b**) B02-iso (1.8 µM) sensitizes MDA-MB-231 cells to olaparib upon 10 days of treatment; (**c**) B02-iso (1.8 µM) does not sensitize MCF10A cells to olaparib upon 10 days of treatment; (**d**) RAD51 knockdown sensitizes MDA-MB-231 cells to olaparib. MDA-MB-231 cells were transfected with either scrambled siRNA or anti-RAD51 siRNA_A and treated with olaparib for 10 days after transfection; (**e**) Levels of RAD51 72 h after transfection with scrambled (scr) or anti-RAD51 siRNA_A (si_A, Origene); (**f**) 1 h MMS treatment sensitizes MDA-MB-231 cells to olaparib alone or in combination with B02-iso (2 µM) during a 4-day treatment; (**g**) 1 h MMS treatment does not sensitize MDA-MB-231 cells to B02-iso during a 4-day treatment. Experiments were repeated at least 3 times, and representative graphs are shown. Error bars indicate the SEM.

PARP inhibitors (PARPi) were developed against tumors deficient in HR, mainly carrying BRCA1/BRCA2 germline mutations [29]. While PARPi are generally inefficient against HR-proficient tumors, it was shown that inhibiting the HR pathway with RAD51 shRNA [51] or with small-molecule inhibitors disrupting the RAD51/BRCA2 interface [52] sensitized cancer cells to PARPi. Here, we tested the effect of B02-iso on the sensitivity of HR-proficient MDA-MB-231 breast cancer cells to PARPi olaparib. The effect of a combination of B02-iso with olaparib on cell viability was tested and compared to the effect of olaparib only. We found that in the presence of 1.8 µM of B02-iso, which alone at this concentration does not impair cell viability (Figure 7a), the efficiency of olaparib killing increased 2.4-fold during a 10-day treatment; the IC_50_ of olaparib decreased from 8.6 µM (olaparib only) to 3.6 µM in combination with B02-iso (Figure 7b). In contrast, in normal breast epithelial cells (MCF 10A), B02-iso at 1.8 µM did not decrease cell viability at the same range of olaparib concentrations (Figure 7c). The effect of B02-iso in potentiating olaparib efficiency in MDA-MB-231 cells was similar to that caused by antiRAD51 siRNA. In this case, the sensitivity of MDA-MB-231 cells for olaparib increased approximately 2-fold; the IC_50_ of olaparib decreased from 6.3 to 3.2 µM after transfection with antiRAD51 siRNA_A, as compared with the cells transfected with scrambled siRNA (Figure 7d). The inhibitory effect of siRNA_A on the RAD51 protein level 72 h after transfection is shown in Figure 7e.

Previously, it was shown that treatment with the combination of alkylating agent MMS and PARP inhibitor drastically decreases cell survival [53]. Alkylating agent MMS induces DNA damage that leads to single-strand breaks (SSB), repaired mainly through the Base Excision Repair (BER). Unrepaired DNA lesions may lead to DSBs or stalled replication forks, resolved by Non-Homologous End-Joining (NHEJ) and HR. Therefore, simultaneous inhibition of the BER and HR pathways combined with alkylating agent treatment might be a useful therapeutic strategy against cancer. We tested the effect of B02-iso in combination with olaparib on the viability of MDA-MB-231 cells that were treated with MMS for 1 h. B02-iso was used in 2 µM concentration, which causes less than 5% growth inhibition during a 4-day treatment (Figure 6a). We found that MMS (100 µM) sensitized MDA-MB-231 cells to low concentrations of olaparib; the cell-killing effect was further significantly increased by the addition of a non-toxic dose of B02-iso (2 µM) (Figure 7f). Note that, during a 4-day treatment, the sensitizing effect of B02-iso on olaparib-treated MDA-MB-231 cells was much less pronounced than during a 10-day treatment; moreover, MMS (100 µM) did not sensitize MDA-MB-231 cells to B02-iso in the absence of olaparib, consistent with the requirement of PARP for the repair of MMS-induced DNA lesions (Figure 7g).

## 4. Discussion

Here, we present a new series of small-molecule RAD51 inhibitors developed from B02, described by us earlier [36]. We show that B02-iso, *p*-I-B02-iso and m-Br-B02-iso are substantially stronger inhibitors of HR than the original B02. B02-iso and especially its derivatives bind strongly to RAD51 *in vitro*. Our docking simulations suggest that the B02-iso, similar to B02, occupies the RAD51 dimer interface. Furthermore, we show that B02-iso and p-I-B02-iso efficiently inhibit the growth of MDA-MB-231 breast cancer cells at concentrations that are not toxic for MCF 10A breast epithelial cells. Finally, B02-iso at non-toxic concentrations potentiates the anti-proliferating activity of PARPi olaparib in HR proficient MDA-MB-231 cells.

To increase the efficiency of screening for new B02 derivatives, we substantially modified the DR-GFP assay. The DR-GFP (Direct Repeat-GFP) assay, developed by M. Jasin’s lab [46], remains the most widely used assay to quantitatively measure the HR capacity in human/mammalian cells. The DR-GFP assay measures the HR event that occurs within the DR-GFP reporter construct integrated into the genome. Modifications of the DR-GFP assay allowing for measurements of HR at specific genomic regions have been recently developed [54,55]. To create a DSB, the DR-GFP assay utilizes a sequence-specific endonuclease. This endonuclease-cleaving site can be located within the construct like in the canonical DR-GFP assay or in a selected genomic region as in ASHRA (assay for site-specific HR activity [55]). While the canonical DR-GFP assay uses a rare-cutting endonuclease I-*Sce*I from the yeast *Saccharomyces cerevisiae* mitochondrial genome, site-specific recombination assays employ CRISPR-Cas9 endonuclease directed to a specific genomic locus by gRNA. Analogous to the original DR-GFP assay, a plasmid-carried luciferase reporter-based HR assay was developed in R. Jensen’s lab [56]. All of these assays require a time-consuming step of transfection of an endonuclease-encoding sequence into mammalian cells, which may complicate the screening of multiple compounds. Bindra et al. [45] created a unique chimeric inducible I-*Sce*I construct and integrated it into U-2 OS cells bearing both HR and altNHEJ reporters, based on GFP and red fluorescent protein dsRed, respectively. They named their system U-2 OS EJ-DR. They successfully applied the EJ-DR system for the high-throughput screening of HR and altNHEJ inhibitors [57]. However, in our study, focused on the screening of HR inhibitors, we wanted to increase the accuracy of the assay by eliminating any bleed-through of fluorescence emission due to the partial overlap of spectral profiles of dsRed and GFP. As a result, we created a testing system that solely contains the DR-GFP reporter and a chimeric inducible I-*Sce*I endonuclease in the genome of U-2 OS cells. We integrated an inducible I-*Sce*I (ddSceGR) construct into the genome of U-2 OS cells and isolated a stable ddSceGR expressing clone, which we named U-2 OS IndDR-GFP cell line. Our U-2 OS IndDR-GFP cells have a background GFP fluorescent signal of approximately 2.3%, and the background fluorescence of the same magnitude was present in the U-2 OS EJ-DR cell line. This background fluorescent signal is likely to be caused by the residual activity of ddSceGR present in the cells in the absence of inducers. In the presence of inducers (TA and Shield1), the fraction of GFP-containing cells was increased to 6.6%, similar to the 7.1% observed for the U-2 OS EJ-DR cells. The level of the GFP fluorescence of the IndDR-GFP system in the induced state (with subtracted background) was approximately twice as high as in the canonical DR-GFP in U-2 OS cells, indicating higher sensitivity of the IndDR-GFP. Overall, the IndDR-GFP assay can facilitate the high-throughput screening and development of HR inhibitors that may serve as potential anti-cancer drugs.

Targeting HR is a promising therapeutic avenue because HR is dysregulated and elevated in many cancers [21,58,59]. Dysregulated HR contributes to genomic instability that may lead to oncogenic transformation and disease progression [60,61]. Genomic instability is likely to arise from HR between repeated sequences abundant in the human genome [60]. Elevated and deregulated HR usually correlates with high levels of RAD51 recombinase [20,62]. High levels of RAD51 originate from the activation of its transcription [63] and post-transcriptional modifications [64,65]. Many tumors are p53-negative, and, in the absence of p53, RAD51 transcription is activated [66]. High RAD51 expression leads to the resistance of tumors to chemotherapy with DNA-damaging agents, such as etoposide or cisplatin [62,65]. Elevated levels of RAD51 expression also correlate with a grade of a tumor [22] and poor disease prognosis [67]. In contrast, RAD51 knockdown was shown to increase the sensitivity of cancer cells to DNA-damaging agents [25,68]. Therefore, targeting RAD51 may increase the susceptibility of tumors to chemo and radiotherapy. Indeed, several small-molecule inhibitors of RAD51 have been developed and were shown to sensitize cancer cells to chemotherapy and [35,52,69,70]. Here, we identified a new series of HR inhibitors, B02-iso, that bind to RAD51. Our docking studies show that B02-iso derivatives occupy a pocket of the RAD51 dimer interface. Using IndDR-GFP assay, we showed that B02-iso and its more active derivatives efficiently inhibit HR *in vitro*. We further demonstrated that B02-iso reduces RAD51 foci formation in U-2 OS IndDR-GFP cells. RAD51 foci serve as a biomarker of HR [71,72,73]. Notably, B02-iso did not alter the amount of γ-H2AX foci, indicating that B02-iso is not genotoxic and that its action is specific towards the RAD51-dependent HR pathway. Further studies investigating the mechanism of B02-iso inhibitors action will follow.

The reduction in EdU incorporation in MCF10A cells, coupled with the increased percent of cells in G1 following exposure to B02-iso, suggests that these non-tumor cells respond to RAD51 inhibition with a G1 arrest. In contrast, our data suggest that a defective G1/S checkpoint contributes to the high sensitivity of breast cancer cells (MDA-MB-231) to B02-iso. A defective G1/S checkpoint in the MDA-MB-231 cell line is a likely consequence of a mutated tumor suppressor p53. MDA-MB-231 cells bear the R280K mutation in the p53 DNA-binding domain, which renders p53 defective in transcription activation [74,75], leading to an impairment in G1 arrest [76,77]. The impact of p53 activity on the efficacy of RAD51i is of significant interest, because approximately 50% of all cancers carry p53 mutations [78]. Recently, it was shown that p53 may either promote or antagonize the anti-proliferating effect of B02 alone or in combination with topotecan in retinoblastoma cells depending on whether the BAX (apoptotic) or p21 (G1 cell cycle arrest) pathways predominate in these cells [79].

The synthetic lethality (SL) approach in cancer therapy, which stemmed from the seminal discovery that BRCA-deficient tumors are sensitive to PARP inhibitors (PARPi) [80], came recently into a broader perspective. Applications of PARPi to HR-defective tumors that manifest the BRCA-ness phenotype are now the subject of intense development [29]. Though PARPi are being successfully used in clinic for HR-deficient cancer therapies [81], resistance to PARPi is a common problem. Among the major factors contributing to the PARP resistance are the restoration of HR function and replication fork stability [82,83,84]. Since the RAD51 protein plays major roles in both HR and replication fork restart and reversal [85,86,87,88], RAD51 inhibitors can help to overcome PARP resistance. In addition, it was found that BRCA1-deficient SUM149 cancer stem cells are resistant to PARPi, and this resistance is mediated by high levels of RAD51 [51]. The resistance of SUM149 cancer stem cells to olaparib was reversed with RAD51 knockdown *in vitro* and *in vivo*, indicating a good potential for a combination of PARPi and RAD51i to alleviate PARPi resistance in BRCA-deficient tumors. RAD51 knockdown also sensitized BRCA-proficient MDA-MB-231 and SUM159 cells to olaparib [51], implying that a combination of RAD51i and PARPi can be used as a strategy to target HR-proficient cancers [35,52,70]. In this way, synthetic lethality can be chemically induced by RAD51i. We show here that the sensitivity of BRCA-proficient MDA-MB-231 breast cancer cells to olaparib was significantly increased in the presence of a non-toxic concentration of B02-iso. These results provide new support for the chemically induced synthetic lethality approach to cancer therapy.

## Figures and Tables

**Table 1:** Comparison of the potency of RAD51 inhibitors.

Compound	IC_50_	K_D_
B02	17.70 ± 3.89 µM	14.6 ± 7.8 µM
B02-iso	4.30 ± 0.75 µM	14.6 ± 6.2 µM
p-I-B02-iso	0.72 ± 0.07 µM	1.4 ± 0.6 µM
p-Br-B02-iso	0.80 ± 0.10 µM	ND
m-Br-B02-iso	0.90 ± 0.18 µM	8.4 ± 1.8 µM
m-I-B02-iso	0.86 ± 0.02 µM	ND
o-Cl-B02-iso	5.56 ± 0.49 µM	ND
o-Br-B02-iso	4.60 ± 1.28 µM	ND

**IC_50_**—the efficiencies of HR inhibition obtained in IndDR-GFP assays. **K_D_**—the equilibrium dissociation constants of the direct binding of B02-iso analogs to hRAD51, as judged by surface-plasmon resonance spectrometry (SPR). ± indicates SD. ND—not determined.

## Data Availability

The data presented in this study are available on request from the corresponding author.

## Supplementary Materials

The following are available online at https://www.mdpi.com/article/10.3390/genes12060920/s1, Figure S1: B01 Derivatives with Phenyl/Benzyl Group Modification, Figure S2: Effect of B02 Derivatives with Phenyl group modifications on HR in U-2 OS IndDR-GFP human cells, Figure S3: Scheme of the synthesis of p-formyl-benzyl derivative of B02, Figure S4: Scheme of the synthesis of B02-iso and derivatives (compounds 6a-g), Figure S5: B02-iso does not change the RAD51 expression level in U-2 OS IndDR-GFP human cells; Table S1: B02 Intermediates, Table S2: B02 Analogs
