# Supplementary material for: New RAD51 Inhibitors to Target Homologous Recombination in Human Cells
Source: Genes (Basel). 2021 Jun 16;12(6):920. doi: 10.3390/genes12060920 (PMC8235719; doi:10.3390/genes12060920)
Supplement: Supplementary file 1 [file genes-12-00920-s001.zip › genes-1250252-supplementary.pdf]

Supplementary information

New RAD51 inhibitors to target homologous recombination in human cells

Irina S. Shkundina[1], Alexander A. Gall[2], Alexej Dick[1], Simon Cocklin[1], and Alexander V. Mazin[1,3]

[1]Department of Biochemistry and Molecular Biology, Drexel University College of Medicine, Philadelphia, PA 19102, USA.

[2]IDbyDNA, Bothell, WA 89011, USA.

[3] Department of Biochemistry and Structural Biology, University of Texas Health Science Center, San Antonio, TX 78229, USA

*Correspondence to:
Alexander Mazin, Ph. D.
UT Health San Antonio
7703 Floyd Curl Drive
San Antonio, Texas 78229-3900
(210) 450-3857
mazin@uthscsa.edu

Supplementary methods

Supplementary tables

Supplementary figures

Supplementary references

Supplementary Methods

P-formyl-benzyl derivative of B02 (4-({4-oxo-2-[(E)-2-(pyridin-3-yl)ethenyl]-3,4-dihydroquinazolin-3-yl}methyl)benzaldehyde, Fig. 1S, *compound 10,* Fig. 3S) was synthesized from the *intermediates 7,8,9*. *Intermediate 7* was obtained according to the procedure described in Langhals et al [3]. To obtain *intermediate 9* isatoic anhydride (16.3 g, 0.1 mol) was added to a solution of *intermediate 7* (19 g, 0.105 mol) in acetonitrile (60 ml), and the mixture was heated at reflux for 30 min and left overnight at room temperature. The mixture was evaporated giving *intermediate 8* as a foam, 26 g. Trimethyl orthoformate (150 ml) was added to *intermediate 8* followed by trifluoroacetic acid (15 ml). Mild exothermic reaction was observed, causing the temperature of the reaction mixture to increase to 40 °C, while clear solution was formed. The mixture was stirred at room temperature for 24 h and monitored by LC-MS. The product showed [M+H$^+$] 325. The reaction mixture was evaporated with toluene, treated with saturated sodium bicarbonate and extracted into ethyl acetated, then, washed with saturated sodium chloride and dried over sodium sulfate. Reaction mixture was purified on silica gel column using ethyl acetate and hexanes for elution. The product was obtained as oil, 16.5 g (51%). In order to obtain *compound 10, intermediate 9* (2.4 g, 7.4mmol) was dissolved in acetic acid (26 ml) and treated with sodium acetate (0.5g, 6mmol) followed by treatment with 3-pyridinecarboxaldehyde (1.0 ml, 10.7 mmol). The reaction mixture was stirred under argon at 80 °C for 18 h, evaporated, and the residue was taken into ethyl acetate, washed with saturated sodium bicarbonate, then, with saturated sodium chloride, and dried over sodium sulfate. The extract was evaporated, re-dissolved in 5 ml of acetonitrile and treated with 1 ml of concentrated HCl, then, heated with stirring at 60 °C for 30 min and evaporated. The product was extracted again into ethyl acetate, washed with saturated sodium bicarbonate, then with saturated sodium chloride, and dried over sodium sulfate. The final product was isolated by flash chromatography on silica gel using ethyl acetate with addition of isopropyl alcohol (20:1) for elution. Fractions with [M+H$^+$] 368 were combined providing the *compound 10*, 1.12 g (41%), m.p. 146-152°. ^{1}H NMR, δ: 9.94 (s, 1H, CHO), 8.86 (d, 1H, *J* = 2.1 Hz, Py-H2), 8.55 (dd, 1H, *J* = 4.7, 1.6 Hz, Py-H6), 8.23 (dd, 1H, *J* = 8.0, 1.1 Hz, Py-H4), 8.12 (ddd, 1H, *J* = 8.0, 1.8, 1.8 Hz, H8), 7.92 (d, 1H, *J* = 15.4 Hz, *trans*-olefin-2′), 7.91-7.86 (m, 1H, H6), 7.87 (d, 2H, *J* = 8.2 Hz, CHO-Ar-**H**), 7.77 (d, 1H, *J* = 7.7 Hz, H5), 7.57 (ddd, 1H, *J* = 7.0, 7.0, 1.1 Hz, H7), 7.47 (d, 1H, *J* = 15.4 Hz, *trans*-olefin-1′), 7.45 (dd, 1H, *J* = 7.8, 4.8 Hz, Py-H5), 7.50 (d, 2H, *J* = 8.2 Hz, CHO-Ar-**H**), 5.76 (s, 2H, CH2).

General method of synthesis of the compounds *6 a-g* (B02-iso and derivatives) is shown on Figure 4S. *Intermediates 3a-g* (Fig. 4S) were synthesized by a general procedure published in [1]. In most cases, compounds were precipitated from aqueous mixtures, washed with water, and dried under vacuum. *Intermediates 4a-g* (Fig. 4S) were synthesized by hydrolysis of corresponding nitriles *3a-g* into amides with water in DMSO in the presence of sodium percarbonate following modified procedure described in [2]. Precisely, solution of nitrile *3a* (2.08g,10 mmol) in 15 ml DMSO was heated to 80 °C. Then, solid sodium percarbonate (20-30% H_2O_2, 3.84 g, 24 mmol) was added to the solution of nitrile followed by small portions of water (total 4 ml) while maintaining the temperature at 80 °C. Reaction mixture was stirred at 80 °C for 4 h, and the product was precipitated by addition of water (60 ml). The product *4a* was filtered, washed with water, dried under vacuum, and taken to the next step without further purification. Other amides were synthesized similarly. *Intermediates 5a-g* (Fig. 4S) were synthesized according the following general procedure. For synthesis of the *intermediate 5g* 20 ml of trifluoroacetic acid was added to a stirred mixture of intermediate *4g* (28.87 g, 82 mmol) and 200 ml of trimethyl orthoformate. Mild exothermic reaction was observed upon

addition, causing the temperature of the reaction mixture to increase to 40 °C, while clear solution was formed. The mixture was stirred at room temperature for 3 h and monitored by LC-MS. Single product with $[M+H^+]$ 377 was formed. 100 ml of water was added followed by 200 ml of saturated sodium bicarbonate. The product was initially precipitated as oil, but it turned into a solid during sonication and could be filtered. The solid was dried on filter under stream of nitrogen and dissolved in boiling xylenes (400 ml), then, cooled to room temperature and stirred overnight to complete crystallization. The product was obtained as a solid, 23.4 g. Mother liquor produced additional pure compound, 3.3 g. Other intermediates, *5a-f*, were obtained similarly. Compounds *6a-g* (Fig. 4S) were synthesized according the following general procedure. To obtain compound *6g* 3-pyridinecarboxaldehyde (1.5 ml, 16 mmol) was added to a solution of intermediate *5g* (3.76 g, 10 mmol) in warm ethylene glycol (20 ml) and sodium acetate (1.56 g, 19 mmol). The reaction mixture was stirred under argon at 90 °C for 9 h. The product precipitated from the reaction mixture as a solid. 20 ml of water was added, the product was filtered, washed with water and dried under vacuum. The product can be recrystallized from acetonitrile giving *6g* (*1-[(4-iodophenyl)methyl]-2-[(E)-2-(pyridin-3-yl)ethenyl]-1,4-dihydroquinazolin-4-one*, 3.21 g) with the m. p.: 275-276°C. Compounds *6a* (*1-benzyl-2-[(E)-2-(pyridin-3-yl)ethenyl]-1,4-dihydroquinazolin-4-one*, m.p. 248-250°), *6b* (*1-[(2-chlorophenyl)methyl]-2-[(E)-2-(pyridin-3-yl)ethenyl]-1,4-dihydroquinazolin-4-one*, m.p. 180-183°), *6c* (*1-[(2-bromophenyl)methyl]-2-[(E)-2-(pyridin-3-yl)ethenyl]-1,4-dihydroquinazolin-4-one*, m.p. 209-212°), *6d* (*1-[(3-bromophenyl)methyl]-2-[(E)-2-(pyridin-3-yl)ethenyl]-1,4-dihydroquinazolin-4-one*, m.p. 210-216°), *6e* (*1-[(4-bromophenyl)methyl]-2-[(E)-2-(pyridin-3-yl)ethenyl]-1,4-dihydroquinazolin-4-one*, m.p. 283-284°) and *6f* (*1-[(3-iodophenyl)methyl]-2-[(E)-2-(pyridin-3-yl)ethenyl]-1,4-dihydroquinazolin-4-one*, m.p. 232-235°) were obtained similarly.

Supplementary tables

Table 1. B02 intermediates

Compound	Yield, %	LCMS	
		$M+H^+$	$M-H^+$
3a	57 (EtOAc - Hexanes)	209	207
3b	60 (EtOAc - Hexanes)	243	ND
3c	91	287, 289	ND
3d	90	287, 289	ND
3e	98	287, 289	ND
3f	57	ND	333
3g	96	ND	333
4a	53	227	225
4b	95	261	ND
4c	69	ND	302, 304
4d	64	ND	302, 304
4e	71	ND	302, 304
4f	84	353	ND
4g	95	353	ND
5a	55 (BuOAc)	251	ND
5b	40 (EtOAc)	285	ND
5c	76 (BuOAc - DMF, 20:1)	329, 331	ND
5d	75 (BuOAc - DMF, 3:1)	329, 331	ND
5e	68 (BuOAc)	329, 331	ND
5f	83 (Xylenes)	377	ND
5g	87 (Xylenes)	377	ND

In parentheses: indicated the solvent systems used for recrystallization. BuOAc – butyl acetate, EtOAc – ethyl acetate;

Table 2. B02 analogs

Compound, Ar	Yield, %	LCMS		^{1}H-NMR, δ, ppm
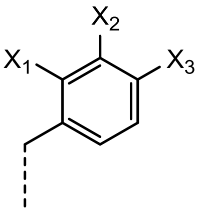		M+H$^+$	M-H$^+$	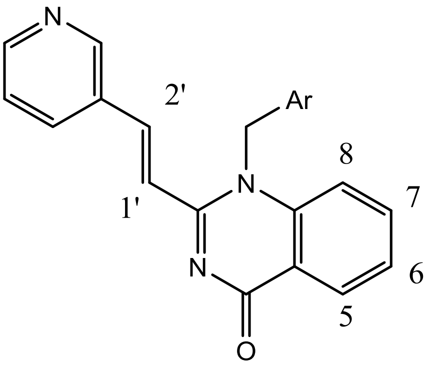
6a<br>1-benzyl-2-[(E)-2-(pyridin-3-yl)ethenyl]-1,4-dihydroquinazolin-4-one<br>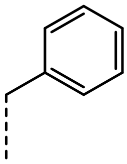	53<br>(BuOAc)	340	338	8.94 (d, 1H, *J* = 1.9 Hz, Py-H2), 8.58 (dd, 1H, *J* = 4.7, 1.4 Hz, Py-H6), 8.22 (ddd, 1H, *J* = 8.0, 1.7, 1.7 Hz, Py-H4), 8.14 (dd, 1H, *J* = 7.8, 1.5 Hz, H5), 8.13 (d, 1H, *J* = 15.1 Hz, *trans*-olefin-2′), 7.74 (ddd, 1H, *J* = 8.6, 7.1, 1.5 Hz, H7), 7.65 (d, 1H, *J* = 8.7 Hz, H8), 7.60 (d, 1H, *J* = 15.1 Hz, *trans*-olefin-1′), 7.50 (t, 1H, *J* = 7.4 Hz, H6), 7.46 (dd, 1H, *J* = 7.9, 4.8 Hz, Py-H5), 7.39-7.34 (m, 2H, Ar-H), 7.31-7.22 (m, 3H, Ar-H), 5.86 (s, 2H, CH2).
6b<br>1-[(2-chlorophenyl) methyl]-2-[(E)-2-(pyridin-3-yl)ethenyl]-1,4-dihydroquinazolin-4-one<br>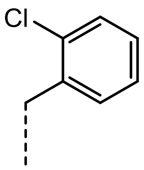	60<br>(Xylenes)	374	372	8.94 (s, 1H, Py-H2), 8.58 (d, 1H, *J* = 3.5 Hz, Py-H6), 8.22 (d, 1H, *J* = 7.4 Hz, Py-H4), 7.8-7.55 (m, 4H, H5, H7, H8, Cl-Ar-H), 8.14 (d, 1H, *J* = 14.7 Hz, *trans*-olefin-2′), 7.54-7.1 (m, 4H, H6, Py-H5, Cl-Ar-H) 7.49 (d, 1H, *J* = 14.7 Hz, *trans*-olefin-1′), 6.90 (d, 1H, *J* = 7.4 Hz Cl-Ar-H), 5.83 (s, 2H, CH2).
6c<br>1-[(2-bromophenyl)methyl]-2-[(E)-2-(pyridin-3-yl)ethenyl]-1,4-dihydroquinazolin-4-one	77<br>(BuOAc-DMF, 4:1)	418, 420	ND	8.94 (d, 1H, *J* = 2.0 Hz, Py-H2), 8.58 (dd, 1H, *J* = 4.6, 1.5 Hz, Py-H6), 8.21 (ddd, 1H, *J* = 8.0, 1.7, 1.7 Hz, Py-H4), 8.15 (dd, 1H, *J* = 7.8, 1.5 Hz, H5), 8.14 (d, 1H, *J* = 15.1 Hz, *trans*-olefin-2′), 7.79 (dd, 1H, *J* = 7.2, 1.8 Hz, H8), 7.74 (ddd, 1H, *J* = 7.8, 7.2, 1.6 Hz, H7), 7.50 (t, 1H, *J* = 7.5 Hz, H6), 7.46 (dd, 1H, *J* = 7.9, 4.8 Hz, Py-H5), 7.47 (d, 1H, *J* =

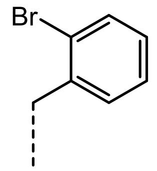				15.1 Hz, *trans*-olefin-1′), (7.32-7.26, m, 3H, Br-Ar-H), 6.88 (dd, 1H, *J* = 6.6, 2.7 Hz, Br-Ar-H), 5.74 (s, 2H, CH2).
6d<br>1-[(3-bromophenyl)methyl]-2-[(E)-2-(pyridin-3-yl)ethenyl]-1,4-dihydroquinazolin-4-one<br>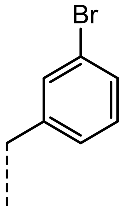	91<br>(BuOAc-DMF, 5:1)	418, 420		8.96 (d, 1H, *J* = 2.0 Hz, Py-H2), 8.59 (dd, 1H, *J* = 4.7, 1.5 Hz, Py-H6), 8.24 (ddd, 1H, *J* = 8.0, 1.8, 1.8 Hz, Py-H4), 8.15 (dd, 1H, *J* = 7.8, 1.5 Hz, H5), 8.13 (d, 1H, *J* = 15.1 Hz, *trans*-olefin-2′), 7.76 (ddd, 1H, *J* = 7.9, 7.2, 1.6 Hz, H7), 7.62 (d, 1H, *J* = 8.6, Hz, H8), (7.60-7.54, m, 2H, Br-Ar-H), 7.50 (t, 1H, *J* = 7.5 Hz, H6), 7.47 (dd, 1H, *J* = 7.9, 4.8 Hz, Py-H5), 7.57 (d, 1H, *J* = 15.1 Hz, *trans*-olefin-1′), (7.30, t, 1H, *J* = 8.0 Hz, Br-Ar-H), 7.13 (d, 1H, *J* = 8.2, Hz, Br-Ar-H), 5.86 (s, 2H, CH2).
6e<br>1-[(4-bromophenyl)methyl]-2-[(E)-2-(pyridin-3-yl)ethenyl]-1,4-dihydroquinazolin-4-one<br>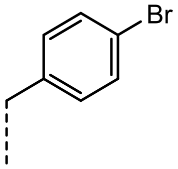	83<br>(BuOAc)	418, 420		8.95 (d, 1H, *J* = 2.1 Hz, Py-H2), 8.59 (dd, 1H, *J* = 4.7, 1.6 Hz, Py-H6), 8.23 (ddd, 1H, *J* = 8.0, 1.8, 1.8 Hz, Py-H4), 8.14 (dd, 1H, *J* = 7.8, 1.6 Hz, H5), 8.13 (d, 1H, *J* = 15.1 Hz, *trans*-olefin-2′), 7.75 (ddd, 1H, *J* = 7.7, 7.1, 1.6 Hz, H7), 7.61 (d, 1H, *J* = 8.6 Hz, H8), 7.57 (d, 1H, *J* = 15.1 Hz, *trans*-olefin-1′), 7.56 (d, 2H, *J* = 8.5 Hz, Br-Ar-H), 7.50 (dd, 1H, *J* = 7.5, 0.6 Hz, H6), 7.47 (dd, 1H, *J* = 8.0, 4.8 Hz, Py-H5), 7.21 (d, 2H, *J* = 8.5 Hz, Br-Ar-H), 5.83 (s, 2H, CH2).
6f<br>1-[(3-iodophenyl)methyl]-2-[(E)-2-(pyridin-3-yl)ethenyl]-1,4-dihydroquinazolin-4-one<br>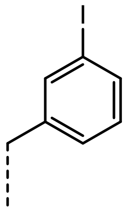	48<br>(BuOAc)	466	ND	8.49 (br. s, 1H, Py-H2), 8.34 (d, 1H, *J* = 4.0, Hz, Py-H6), 8.01 (d, 1H, *J* = 7.6, Hz, Py-H4), 7.72-756 (m, 5H), 7.46-7.38 (m, 3H), (7.25, t, 1H, *J* = 6.0 Hz, I-Ar-H), 7.07 (t, 1H, *J* = 7.5, Hz, I-Ar-H), 6.97 (d, 1H, *J* = 6.8, Hz, I-Ar-H), 5.66 (s, 2H, CH2).

6g<br><br>1-[(4-iodophenyl)methyl]-2-[(E)-2-(pyridin-3-yl)ethenyl]-1,4-dihydroquinazolin-4-one<br><br>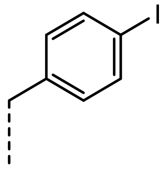	69 (ACN)	466	ND	8.95 (d, 1H, *J* = 2.1 Hz, Py-H2), 8.59 (dd, 1H, *J* = 4.7, 1.5 Hz, Py-H6), 8.23 (ddd, 1H, *J* = 8.0, 1.8, 1.8 Hz, Py-H4), 8.14 (dd, 1H, *J* = 7.8, 1.6 Hz, H5), 8.13 (d, 1H, *J* = 15.1 Hz, *trans*-olefin-2′), 7.77-7.71 (m, 1H, H7), 7.73 (d, 2H, *J* = 8.4 Hz, I-Ar-H), 7.60 (d, 1H, *J* = 8.7 Hz, H8), 7.56 (d, 1H, *J* = 15.1 Hz, *trans*-olefin-1′), 7.50 (dd, 1H, *J* = 7.5, 0.6 Hz, H6), 7.47 (dd, 1H, *J* = 8.0, 4.8 Hz, Py-H5), 7.06 (d, 2H, *J* = 8.4 Hz, I-Ar-H), 5.81 (s, 2H, CH2).

BuOAc - butyl acetate, ACN - acetonitrile

Supplementary figures

o-dichlorophenyl

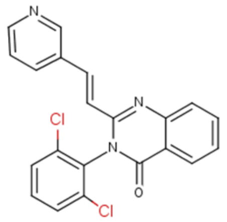

p-chlorophenyl

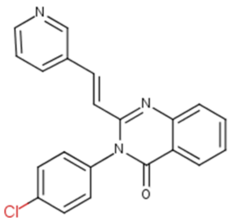

p-bromophenyl

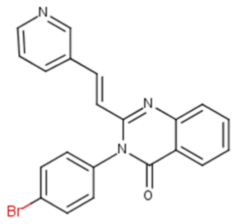

o-methoxyphenyl

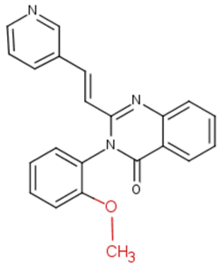

m-methoxyphenyl

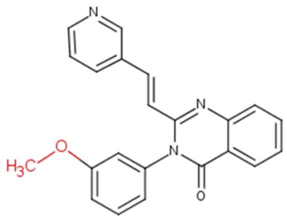

p-methoxyphenyl

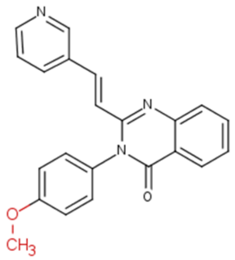

p-hydroxyphenyl

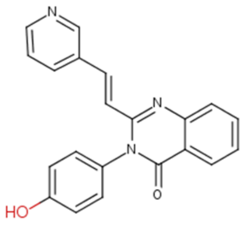

p-hydroxyethyl-phenyl

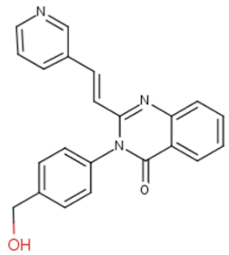

p-formyl-benzyl

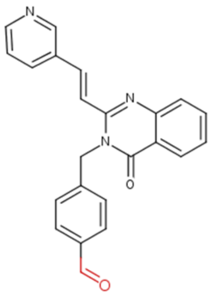

o-methylphenyl

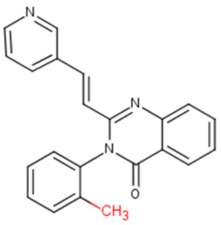

o-methylphenyl (I)

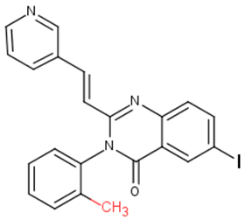

Figure S1. B02 derivatives with phenyl/benzyl group modification

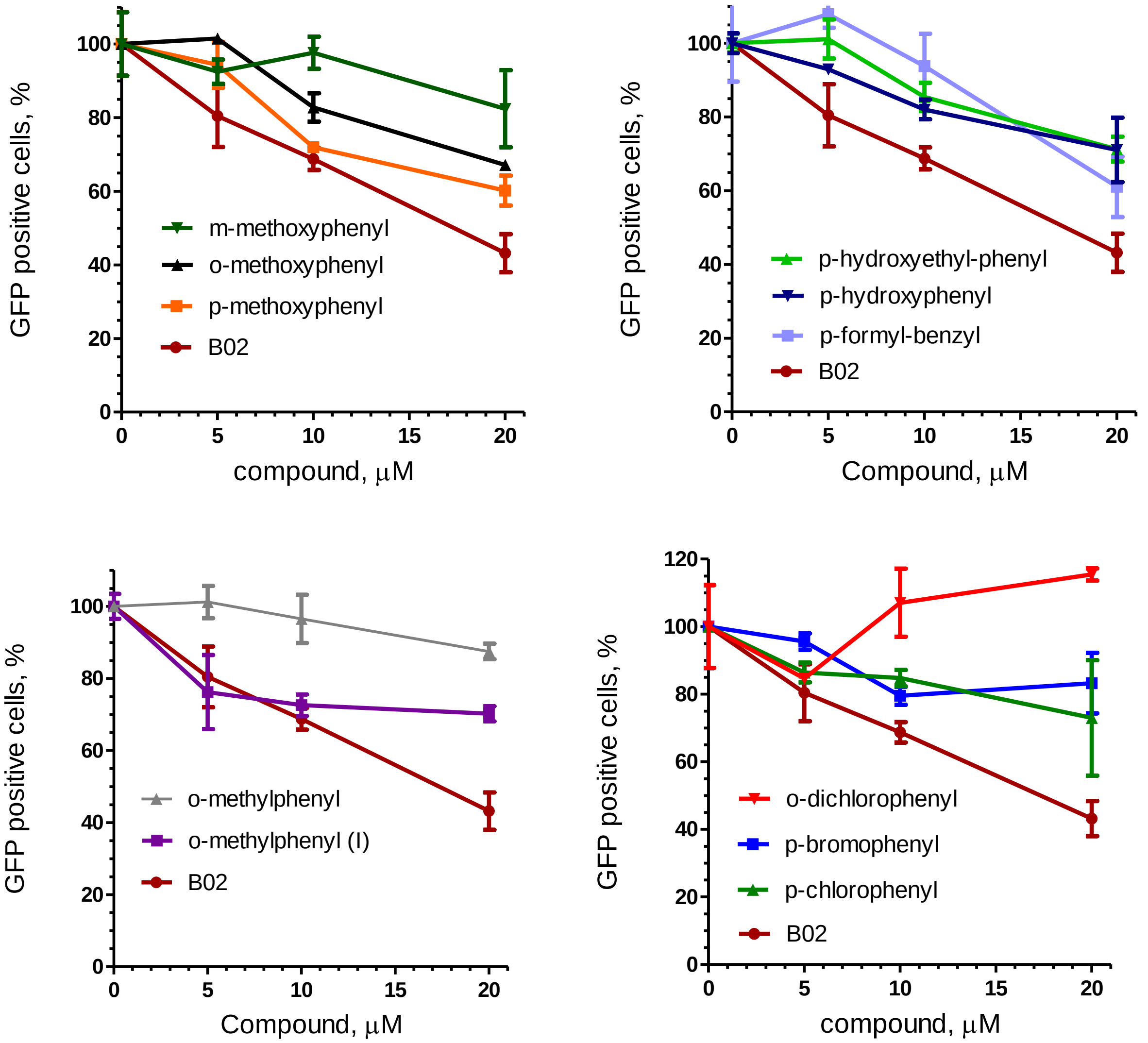

Figure 2S. Effect of B02 derivatives with phenyl group modifications on HR in U-2 OS IndDR-GFP human cells. The HR activity was measured using the IndDR-GFP assay. The yield of GFP positive cells in untreated controls was expressed as 100%. Error bars indicate SEM.

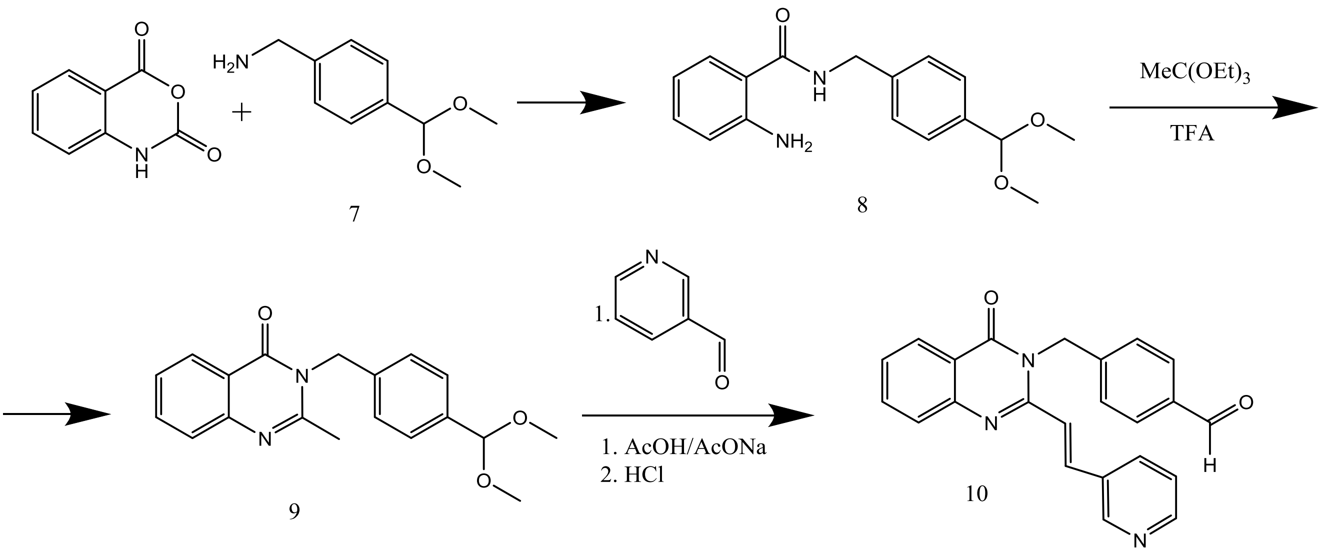

Figure 3S. Scheme of the synthesis of p-formyl-benzyl derivative of B02.
P-formyl-benzyl derivative of B02 (10) was synthesized from the *intermediates 7, 8, 9*

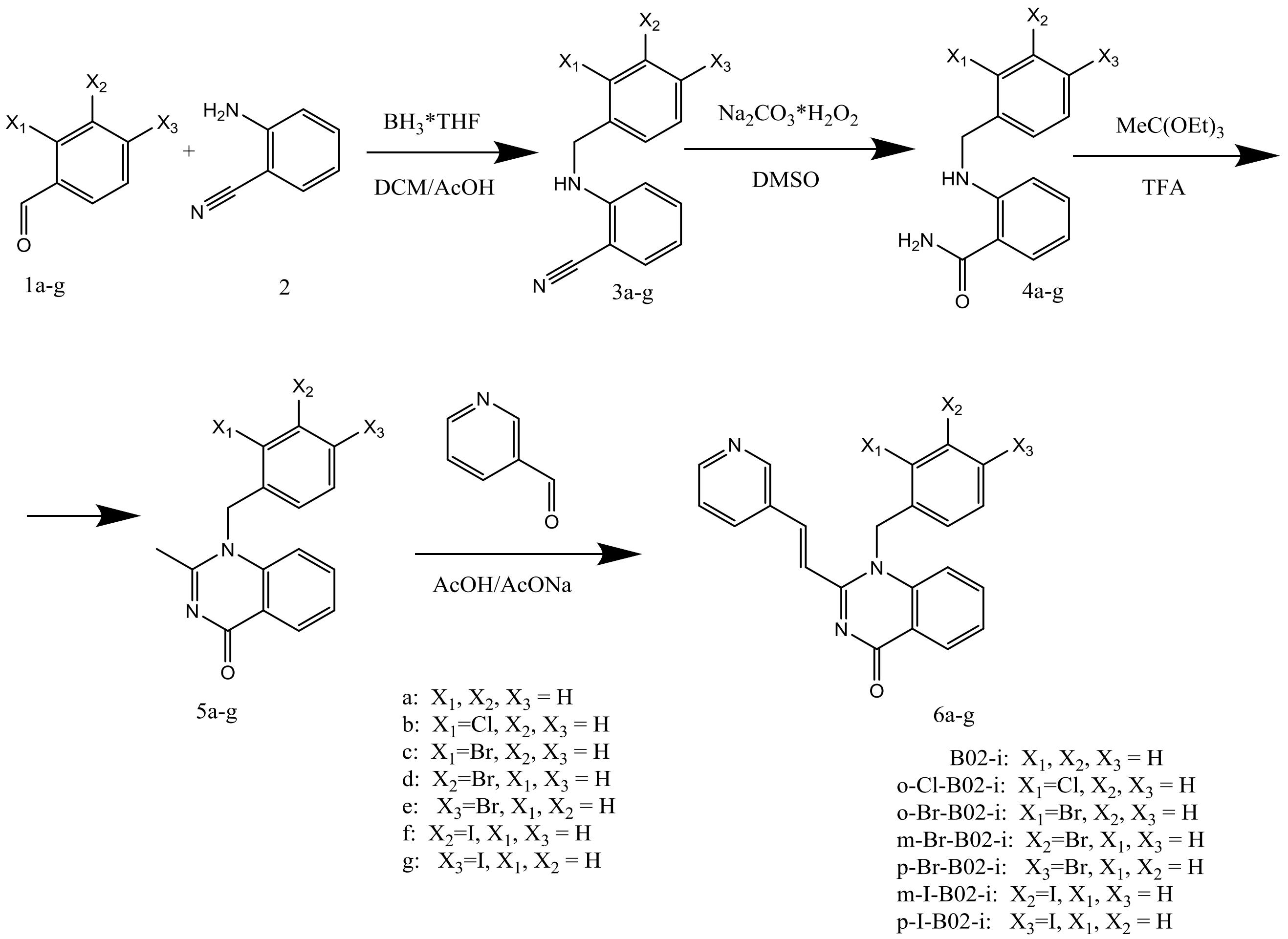

Figure 4S. Scheme of the synthesis of B02-iso and derivatives (compounds *6a-g*)

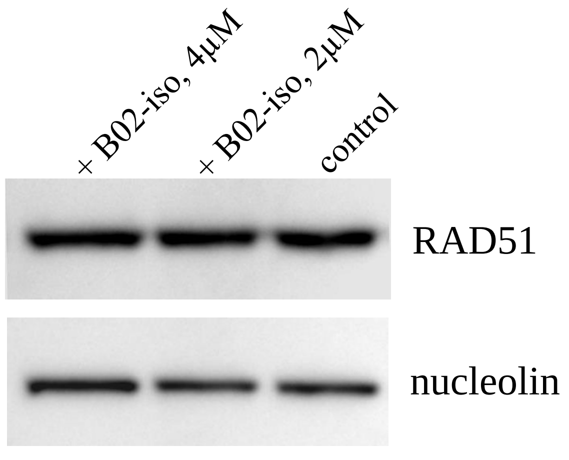

Figure 5S. B02-iso does not change the RAD51 expression level in U-2 OS IndDR-GFP cells. Cell lysates were prepared from U-2 OS IndDR-GFP cells, which were treated with Shield1 and triamcinolone acetonide to induce DNA double-strand breaks and with B02-iso for 24 h. 10 µg of protein lysates were loaded and analyzed by SDS-PAGE. The proteins were transferred to PVDF membranes and probed with anti-RAD51 rabbit polyclonal (1:1000, GeneTex, GTX100469) and anti-nucleolin mouse monoclonal (1:1000, Santa Cruz Biotechnology, sc-17826) antibodies.

Supplementary references

1. Pletz, J., B. Berg, and B. Rolf, *A General and Direct Reductive Amination of Aldehydes and Ketones with Electron-Deficient Anilines.* . Synthesis, 2016. **48**(09): p. 8.
2. Kabalka, G.W., et al., *The Transformation of Nitriles into Amides Using Sodium Percarbonate.* Synthetic Communications, 2006. **20**(10): p. 1445-1451.
3. Langhals, H., et al., *The fluorescence labelling of primary amines with perylenetetracarboxdiimides.* European Journal of Organic Chemistry, 2007. **2007**(26): p. 4328 - 4336.
